# Inflammation-Associated Microsatellite Alterations Caused by MSH3 Dysfunction Are Prevalent in Ulcerative Colitis and Increase With Neoplastic Advancement

**DOI:** 10.14309/ctg.0000000000000105

**Published:** 2019-11-26

**Authors:** Koji Munakata, Minoru Koi, Takahito Kitajima, Stephanie Tseng-Rogenski, Mamoru Uemura, Hiroshi Matsuno, Kenji Kawai, Yuki Sekido, Tsunekazu Mizushima, Yuji Toiyama, Takuya Yamada, Masayuki Mano, Eiji Mita, Masato Kusunoki, Masaki Mori, John M. Carethers

**Affiliations:** 1 Division of Gastroenterology, Departments of Internal Medicine and Human Genetics, Rogel Cancer Center, University of Michigan, Ann Arbor, Michigan, USA;; 2 Department of Surgery, National Hospital Organization, Osaka National Hospital, Osaka, Japan;; 3 Department of Gastroenterological Surgery, Osaka University, Osaka, Japan;; 4 Department of Gastrointestinal and Pediatric Surgery, Mie University, Tsu, Japan;; 5 Department of Gastroenterology and Hepatology, National Hospital Organization, Osaka National Hospital, Osaka, Japan;; 6 Department of Pathology, National Hospital Organization, Osaka National Hospital, Osaka, Japan.

## Abstract

**OBJECTIVES:**

Inflammation-associated microsatellite alterations (also known as elevated microsatellite alterations at selected tetranucleotide repeats [EMAST]) result from IL-6–induced nuclear-to-cytosolic displacement of the DNA mismatch repair (MMR) protein MSH3, allowing frameshifts of dinucleotide or longer microsatellites within DNA. MSH3 also engages homologous recombination to repair double-strand breaks (DSBs), making *MSH3* deficiency contributory to both EMAST and DSBs. EMAST is observed in cancers, but given its genesis by cytokines, it may be present in non-neoplastic inflammatory conditions. We examined ulcerative colitis (UC), a preneoplastic condition from prolonged inflammatory duration.

**METHODS:**

We assessed 70 UC colons without neoplasia, 5 UC specimens with dysplasia, 14 UC-derived colorectal cancers (CRCs), and 19 early-stage sporadic CRCs for microsatellite instability (MSI) via multiplexed polymerase chain reaction capable of simultaneous detection of MSI-H, MSI-L, and EMAST. We evaluated UC specimens for MSH3 expression via immunohistochemistry.

**RESULTS:**

UC, UC with dysplasia, and UC-derived CRCs demonstrated dinucleotide or longer microsatellite frameshifts, with UC showing coincident reduction of nuclear MSH3 expression. No UC specimen, with or without neoplasia, demonstrated mononucleotide frameshifts. EMAST frequency was higher in UC-derived CRCs than UC (71.4% vs 31.4%, *P* = 0.0045) and higher than early-stage sporadic CRCs (66.7% vs 26.3%, *P* = 0.0426). EMAST frequency was higher with UC duration >8 years compared with ≤8 years (40% vs 16%, *P* = 0.0459).

**DISCUSSION:**

Inflammation-associated microsatellite alterations/EMAST are prevalent in UC and signify genomic mutations in the absence of neoplasia. Duration of disease and advancement to neoplasia increases frequency of EMAST. MSH3 dysfunction is a potential contributory pathway toward neoplasia in UC that could be targeted by therapeutic intervention.

## INTRODUCTION

Defects in DNA mismatch repair (MMR) function are associated with human cancer development and progression (1–3). Germline mutations in the MMR genes *MSH2*, *MLH1*, *PMS2*, and *MSH6* are causal for Lynch syndrome, an inherited condition in which patients may develop colorectal cancer (CRC) (constituting ∼3% of all CRCs) and cancers of the rest of the gastrointestinal tract, female reproductive and urinary tracts, and specific skin and CNS tumors (4). Somatic inactivation of *MLH1* via biallelic hypermethylation of *MLH1*'s promoter is observed in ∼15% of sporadic CRCs, and biallelic somatic mutations of *MSH2*, *MLH1*, *PMS2*, or *MSH6* are the apparent cause for Lynch-like syndrome in 1%–2% of all patients with CRC (5). The common finding in these patients with germline or somatic MMR deficiency is the presence of microsatellite instability–high (MSI-H) in tumor DNA, defined by an NCI Consensus Panel as at least 2 frameshift mutations identified with the use of a panel of at least 5 mono- and dinucleotide microsatellite markers (6). In general, the outcome of patients with an MSI-H tumor is better than a patient with a microsatellite stable (MSS) tumor, and patients with MSI-H tumors can benefit with further increased survival through the use of immune checkpoint inhibitors due to hypermutated cancer genomes that drive immune-responsive neoantigens generated from exon coding mononucleotide microsatellite frameshifts (7,8). Another form of MSI is termed elevated microsatellite alterations at selected tetranucleotide repeats (EMAST) that has been observed in a variety of cancers including CRCs (9,10). EMAST (in the absence of MSI-H) is caused by an IL-6–induced nuclear-to-cytosol shift of the MMR protein MSH3, triggering subsequent di-, tri-, and tetranucleotide and longer frameshifts of genomic microsatellites in the nucleus (11,12). EMAST is observed in ∼50% of all CRCs (2,7). Patients with EMAST CRC, unlike patients with MSI-H CRC, demonstrate poor survival compared with patients without EMAST CRC and have advanced-stage disease and frequent metastasis (13–15). Because the mechanism of MSH3 dysfunction is due to intracellular displacement of MSH3 by proinflammatory cytokine signaling, EMAST can also be termed inflammation-associated microsatellite alterations. This term differentiates the isolated MSH3 dysfunction observed in inflammation-associated microsatellite alterations from secondary *MSH3* mutations as a result of *MLH1* deficiency in sporadic CRCs, a scenario where a tumor would manifest mononucleotide frameshifts in addition to di-, tri-, and tetranucleotide instability (9,16,17). In particular, tumors defective for *MLH1*, *MSH2*, or *PMS2* would demonstrate mono-, di-, tri-, and tetranucleotide frameshifts, whereas tumors defective for *MSH6* would manifest mostly mono- and some dinucleotide frameshifts, and tumors with isolated *MSH3* dysfunction would demonstrate di-, tri-, and tetranucleotide instability and no mononucleotide frameshifts (9). With *MSH3* deficiency, affected cells will experience a defect in MMR function and increased DNA double-strand breaks (DSBs) that can lead to aneuploidy and loss of heterozygosity (LOH) events, generating a complex DNA repair deficit (10,15,18,19). One or both of these DNA repair deficits may contribute to the pathogenesis of tumors, including metastases (15).

The observations of MSI-H and EMAST have largely been among cancers (10,20). Noncancer tissues, presumably due to intact MMR function and stable normal genomes, generally do not manifest MMR deficiency. Rarely have MMR defects been found in noncancer tissue (21). We have previously observed evidence for MSH3 dysfunction within hamartomatous polyp epithelium, whose polyp is a non-neoplastic lesion that possesses an expanded inflammatory lamina propria (22). With this last observation coupled with the recent characterized mechanism of proinflammatory cytokine-induced MSH3 intracellular displacement, we sought out any evidence of MSH3 dysfunction in ulcerative colitis (UC), an inflammatory bowel disease condition in which tissues contain several proinflammatory cytokines including IL-6. UC can progress to CRC in some patients, with the greatest risk factor being disease duration over 8 years (23–26), but also include the extent of UC, presence of primary sclerosing cholangitis, family history of sporadic CRC, severity of bowel inflammation, and young age at onset for UC. Previous reports have examined MSI using mono- and dinucleotide markers on UC specimens with varied results, but generally conclude that UC and UC-associated neoplasia are not commonly an MSI-H–driven process (27–30). Some reports show that UC specimens lack mononucleotide instability but occasionally possess dinucleotide instability, which raises the possibility of isolated MSH3 dysfunction (31–34). Several reports have hypothesized because there is little evidence for loss of MMR protein expression in UC that the repeated bombardment of tissue by inflammation might alter MMR function in the absence of mutation or loss of protein (35–40). Here, we examined whether our previous discovery of inflammation-associated microsatellite alterations and MSH3 dysfunction among CRCs is operative in non-neoplastic but inflammatory UC. Such finding would extend the role of inflammation as a continuing cause of DNA damage before any neoplastic transformation.

## METHODS

### Cell lines

The human colon cancer cell lines HCT116+3+5 (proficient in MSH3), G5 (deficient in MSH3), and DLD1 (deficient in MSH6) have been described previously (41). HCT116+3+5 and DLD1 were grown in Dulbecco Modified Eagle Medium supplemented with 10% fetal bovine serum. G5 cells were maintained in Dulbecco Modified Eagle Medium with 10% fetal bovine serum and 0.6 μg/mL of puromycin.

### Clinical samples

A total of 70 human UC clinical tissue samples, 14 UC-derived CRC tissue samples, and 5 UC with dysplasia tissue samples were collected from Osaka University Hospital and Mie University Hospital. Fifty-six of the 70 patients with UC had adequate demographic clinical information, such as age, sex, disease duration, and types of UC (pancolitis or left-sided colitis) and limited data on patient therapy usage (some data on steroid and 5-ASA usage and little or no data on use of azathioprine, 6-mercaptopurine, and biologic therapies such as anti–TNF-α, vedolizumab, and tofacitinib). Although we do not have the presurgery Mayo Disease Activity Index scores for the 70 patients with UC, all patients had disease severe enough to warrant surgery (colectomy) based on clinical practice because this group did not have dysplasia or cancer. Nineteen early-stage CRC (AJCC stage 0 and I) samples were collected from Osaka National Hospital. Ten normal human colon tissues were collected from Osaka University Hospital as controls. These patients did not have UC and had partial resections for reasons such as obstruction, volvulus, and diverticular disease. All institutions had IRB approval to conduct this study. All UC-related samples were from archived formalin-fixed, paraffin-embedded specimens taken from the colectomies that were performed.

### DNA extraction

All epithelial tissues were microdissected from paraffin-embedded 10-μm tissue sections. DNA was isolated and purified from the microdissected tissues as previously described (42). DNA from noninflamed submucosa and/or serosa tissues were used as controls.

### Multiplex polymerase chain reaction and determination of MSI and EMAST

We established a new multiplex polymerase chain reaction (PCR) combining 14 microsatellite markers in 4 reactions for use in MSI determinations, capable of determining MSI-H, MSI-L, EMAST, and MSS simultaneously. A QIAGEN Multiplex PCR Kit (QIAGEN, Hilden, Germany) was used according to the manufacturer's instructions. The cycling condition was as following: 95 °C for 10 minutes for initial heat activation; 45–50 cycles of 94 °C for 30 seconds, 55 °C for 30 seconds, and 72 °C for 30 seconds; and a final extension at 72 °C for 40 minutes. Cycling conditions were altered to 52 °C for 30 seconds for amplification of D2S123, D5S346, D17S250, and MYCL1. Primer sequences are listed in the Table, Supplementary Digital Content 1, http://links.lww.com/CTG/A129. We used 2 mononucleotide (*BAT25* and *BAT26*), 5 dinucleotide (*D2S123*, *D5S346*, *D17S250*, *D18S64*, and *D18S69*), and 7 tetranucleotide microsatellite sequences (*D9S242*, *D20S82*, *D20S85*, *D19S394*, *D8S321*, *MYCL1*, and *RBM47*) as previously described (11,15,42). We defined EMAST as at least 1 tetranucleotide marker with frameshift without any frameshift of a mononucleotide marker and combined this group with MSI-L samples due to both MSI-L and EMAST being caused by MSH3 dysfunction (15,43).

### Western blot analysis

Whole-cell protein was extracted from cell lines and separated by sodium dodecylsulfate-polyacrylamide gel electrophoresis gel. The separated proteins were transferred to nitrocellulose membranes. Anti-human MSH6 mouse monoclonal antibody (clone 44, Cat No. 610919, dilution: 1:500; BD Biosciences, San Jose, CA), anti-human MSH3 mouse monoclonal antibody (clone 52, Cat No. 611390, dilution: 1:500; BD Biosciences), anti-human MSH2 mouse monoclonal antibody (clone G219-1129, Cat No. 556349, dilution: 1:200; BD Biosciences), anti-human MLH1 mouse monoclonal antibody (clone G168–728, Cat No. 554073, dilution: 1:500; BD Biosciences), anti-human PMS2 mouse monoclonal antibody (clone A16–4, Cat No. 556415, dilution: 1:500; BD Biosciences), and anti–α-tubulin antibody (dilution 1:8,000; Sigma-Aldrich, St. Louis, MO) were used as primary antibodies for the detection of specific proteins. Goat anti-mouse antibody (dilution: 1:1,000; Cell Signaling Technology, Danvers, MA) was used as a secondary antibody. Signals were detected using SuperSignal West Pico (Thermo Scientific, Waltham, MA) and captured by an ImageQuant LAS4000 system (GE Healthcare, Chicago, IL).

### Immunohistochemistry

Immunohistochemical staining was performed using 5-μm-thick paraffin-embedded sections on the 10 normal colons collected and 10 randomly selected UC colons out of 70 collected. After deparaffinization in xylene and dehydration in graded ethanol solutions, tissue sections were heated at 121 °C for 15 minutes in TE9 buffer (pH 9.0). Slides were incubated with 3% hydrogen peroxide for 20 minutes to quench endogenous peroxide activity. Samples were washed with PBS and then incubated with immunohistochemistry (IHC) blocking diluent (Leica Biosystems, Wetzlar, Germany) for 20 min, followed by incubation with primary antibody at 4 °C overnight. After washing with PBS, the sections were incubated with secondary antibody for 30 minutes. Samples were developed using 3,3-diaminobenzidine tetrahydrochloride and contrasted using hematoxylin. Anti-human MSH3 rabbit monoclonal antibody (clone EPR4334 (2), Cat No. ab111107, dilution: 1:1,000; Abcam, Cambridge, MA), anti-human MSH6 rabbit monoclonal antibody (clone EPR3945, Cat No. GTX62383, dilution: 1:200; GeneTex, Irvine, CA), and anti-human IL-6 mouse monoclonal antibody (Cat No. ab9324, dilution: 0.25 μg/mL; Abcam) were used as primary antibodies.

### Statistical analysis

The StatView 5.0 program (Abacus Concepts, Piscataway, NJ) was used for statistical analysis, Student *t* test, Fisher exact test, Mann-Whitney *U* test, and 2 × 2 contingency table analyses. *P* < 0.05 indicated a significant difference.

## RESULTS

### Multiplexed PCR accurately determines MSI and EMAST status and confirms MSH3 deficiency as cause of EMAST

**Figure 1.**
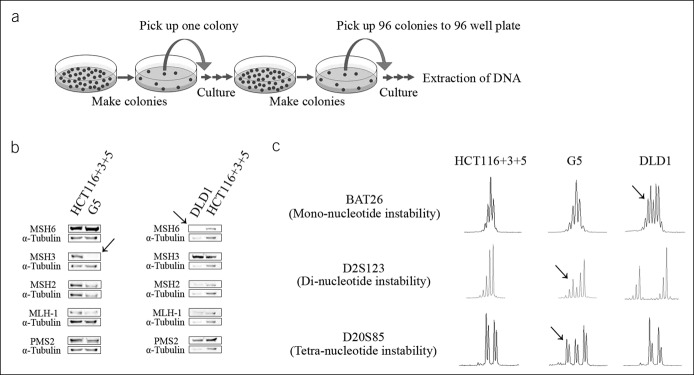
Cloning, mismatch repair protein expression, and fragment analysis for MSI in colorectal cancer cells. (**a**) Single cell cloning methodology was performed twice in series for single cell purification. (**b**) DNA mismatch repair protein expression in HCT116+3+5, G5, and DLD1 cells. G5 cells lack MSH3 protein expression, and DLD1 cells lack MSH6 protein expression. α-Tubulin was used as a loading control. (**c**) Examples of fragment analysis at the mononucleotide microsatellite *BAT26*, the dinucleotide microsatellite *D2S123*, and the tetranucleotide microsatellite *D20S85* from the DNA of HCT116+3+5, G5, and DLD1 cells. Note frameshift mutations (arrows) at *BAT26* for *MSH6*-defective DLD1 cells and frameshift mutations at *D2S123* and *D20S85* for *MSH3*-defective G5 cells.

We set out to create a multiplexed PCR method for detecting all forms of MSI, capable of determining MSI-H, MSI-L, EMAST, and MSS efficiently and simultaneously. Furthermore, we wanted to confirm previous observations that deficiency in MSH3 coincides and causes EMAST (10,12,44). We developed primers to successfully amplify 2 mononucleotide, 5 dinucleotide, and 7 tetranucleotide microsatellite markers that have been individually previously used for detection of MSI and EMAST (see Table, Supplementary Digital Content 1, http://links.lww.com/CTG/A129) in a multiplexed fashion using 4 groups of PCR reactions followed by fragment analyses (see Figure, Supplementary Digital Content 2, http://links.lww.com/CTG/A130, which shows example fragment chromatograms of the 14 markers). We then used cell lines in which the *MSH3* and *MSH6* status was known to assess microsatellite frameshifts in the presence or absence of those DNA MMR genes. Cell lines were cloned, and each isolated cloned colony was subject to the multiplex PCR (Figure 1a), enabling a purity that is often absent from the heterogeneity of a tumor (45). HCT116+3+5 cells are derived from *MLH1*- and *MSH3*-deficient parental HCT116 CRC cells where 1 copy of human chromosome 3 (correcting *MLH1* deficiency) and 1 copy of human chromosome 5 (correcting *MSH3* deficiency) have been transferred (41). Western blots confirm expression of MLH1 and MSH3 in this cell line (Figure 1b). The cell line G5 is derived from HCT116+3+5 cells by transfection of a tetracycline-regulated retroviral vector that encodes shRNA against MSH3 (41). Western blots confirm acquired absence of MSH3 expression in G5 cells (Figure 1b). DLD1 CRC cells are known to be *MSH6* deficient (44), and Western blots confirm the absence of MSH6 expression (Figure 1b). Fragment analyses of cell clones demonstrate patterns of MSI based on the presence or absence of *MSH3* and *MSH6* (Figure 1c). Fully MMR-proficient HCT116+3+5 cells demonstrated no mononucleotide frameshifts, no dinucleotide framseshifts, and 1 tetranucleotide frameshift among 96 cell clones (Table 1). In contrast, *MSH3*-deficient G5 cell clones show no mononucleotide frameshifts but possess excessive dinucleotide and tetranucleotide frameshifts (Table 1). *MSH6*-deficient DLD1 cell clones demonstrate excessive mononucleotide frameshifts with 1 dinucleotide frameshift and no tetranucleotide frameshifts (Table 1). Our data confirm that *MSH3* deficiency causes dinucleotide or greater MSI (EMAST and MSI-L), whereas *MSH6* deficiency causes dinucleotide or lesser MSI (MSI-H). Our multiplexed PCR approach is accurate in simultaneously detecting frameshifts at mono-, di-, and tetranucleotide microsatellite markers to allow determination of the MSI and EMAST status of human DNA.

**Table 1.**
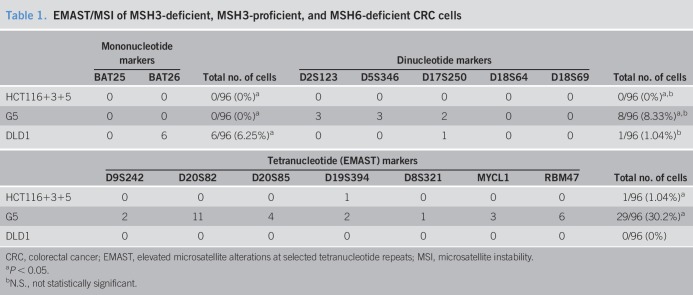
EMAST/MSI of MSH3-deficient, MSH3-proficient, and MSH6-deficient CRC cells

### UC tissues demonstrate reduced MSH3 expression

**Figure 2.**
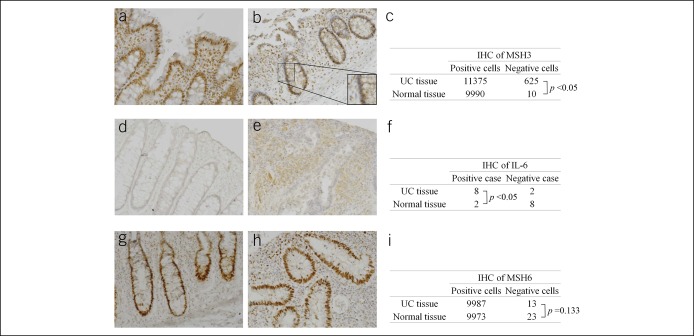
Immunohistochemistry (IHC) expression of MSH3, MSH6, and IL-6 in normal and ulcerative colitis (UC) colon tissues. MSH3 IHC in (**a**) normal and (**b**) UC colon sections, with (**c**) analysis for nuclear expression. IHC for IL-6 expression in (**d**) normal and (**e**) UC colon tissues, with (**f**) analysis for differences in tissue expression. MSH6 IHC in (**g**) normal and (**h**) UC colon tissues, with (**i**) analysis for nuclear expression.

We have previously shown in CRCs and CRC cells that IL-6 signaling triggers a nuclear-to-cytosol displacement of MSH3 (11). This mechanism seems to coincide with loss of nuclear MSH3 expression in CRCs (46,47). Although this phenomenon has largely been seen among cancers that have surrounding inflammation as a source of IL-6, we have previously reported absence of MSH3 expression within the epithelium of hamartomatous polyps, which contain inflammation but are not neoplastic (22). We wondered if the elevated neoplastic risk condition of UC, a type of inflammatory bowel disease whose affected tissues exhibit several proinflammatory cytokines including IL-6, could harbor loss of MSH3 expression similar to our previous findings in CRCs, perhaps as 1 mechanism that could cause DNA damage and elevate neoplastic risk. We performed IHC on 10 randomly selected samples from our 70 resected UC colon specimens and 10 normal colonic tissues for expression of MSH3, IL6, and MSH6 (Figure 2). For MSH3 and MSH6 expression, 1000 cells were counted in 10 separate ×100 microscope fields, yielding total counts of approximately 10,000 cells for which to assess nuclear expression. IHC demonstrated uniform nuclear MSH3 expression in normal colons (Figure 2a) that was reduced and/or heterogeneous in UC colons (Figure 2b). In comparing nuclear MSH3 expression among epithelial cells between UC and normal colons, UC colons showed significant loss of nuclear MSH3 expression in over 5% of epithelial cells (typically in apical portions of crypts) compared with 0.1% for normal colons (Figure 2c). Eight of 10 UC colons showed elevated tissue and lamina propria IL-6 expression compared with 2 of 10 or normal colons (Figure 2d–f). The elevated IL-6 expression among UC colons is consistent with our previous data in CRCs on this being coincident with reduced MSH3 expression. As a control, we also performed IHC for MSH6 expression, which does not undergo nuclear-to-cytosol trafficking by IL-6 signaling (11). As shown in Figure 2g–i, there is no difference in MSH6 expression between normal and UC colon epithelium. Our data demonstrate elevated expression of IL-6 among UC colons that is coincident with reduced MSH3 epithelial expression.

### UC and UC-associated neoplasia demonstrate inflammation-associated microsatellite alterations (EMAST)

With the observation of elevated IL-6 expression that is coupled with reduced MSH3 expression in UC samples, we set out to determine whether the biomarker for absent MSH3 function, EMAST, is present in UC tissues. Such a finding would demonstrate that DNA damage via frameshift mutation would be present in UC, a novel finding. Because of previous findings that the mechanism for MSH3 displacement is due to proinflammatory IL-6 signaling, EMAST that is observed because of isolated MSH3 dysfunction can be termed inflammation-associated microsatellite alterations (17). This term sets it apart from other causes of MSH3 dysfunction such as secondary frameshift mutation of *MSH3* as a result of MLH1 hypermethylation (and would demonstrate both EMAST and MSI-H in the tissue).

We used our multiplexed PCR method on 70 resected UC colons that had no evidence of neoplasia. Among the 70 cases, none showed frameshift mutations in mononucleotide microsatellite markers, 5 cases (7.1%) showed dinucleotide MSI, and 22 cases (31.4%) demonstrated tetranucleotide frameshifts, all consistent with our observed reduced MSH3 expression. This is in contrast to no microsatellite frameshifts among mono-, di-, or tetranucleotide markers for normal colon tissue (Table 2). Thus, even in the absence of neoplasia, UC tissues demonstrate ongoing DNA damage and mutations that are likely due to the presence of proinflammatory cytokines.

**Table 2.**
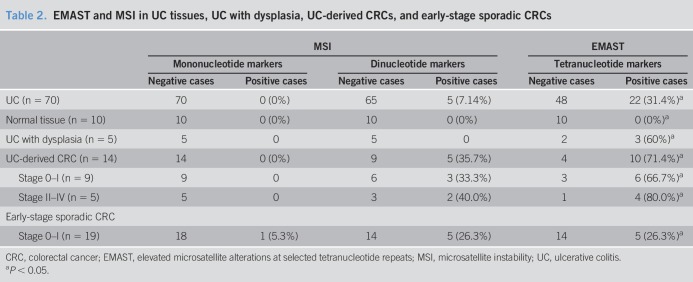
EMAST and MSI in UC tissues, UC with dysplasia, UC-derived CRCs, and early-stage sporadic CRCs

We further examined 5 UC colons with evidence of dysplasia, an important finding and decision point in the clinical care approach from surveillance of patients with UC, as dysplasia is a precancerous condition (24,48). Using our multiplexed PCR method, 3 of 5 UC colons with dysplasia (60%) demonstrated tetranucleotide frameshifts, with no mononucleotide or dinucleotide instability. This again is consistent with isolated MSH3 dysfunction (Table 2).

We wanted to compare UC-derived CRCs with UC without neoplasia and to early-stage sporadic CRCs for inflammation-associated microsatellite alterations. To this end, we performed our multiplexed PCR on 14 resected UC-derived CRCs and demonstrated no mononucleotide instability among the samples, 5 (35.7%) samples with dinucleotide frameshifts, and 10 (71.4%) samples with tetranucleotide frameshifts (Table 2). Again, this is consistent with isolated MSH3 dysfunction, and the frequency of EMAST among UC-derived CRCs was significantly higher than the frequency among UC samples without neoplasia (*P* = 0.0045), suggesting progressive accumulation of frameshifts with advancing tumorigenesis (Table 2). The high frequency of EMAST among UC-derived CRCs is comparable to multiple previous reports of sporadic CRCs, which demonstrate EMAST among 50% of CRCs (13,20). However, our previous report showed that the frequency of EMAST was dependent on CRC stage, with 62% of EMAST CRCs in stage III/IV and 37% of EMAST CRCs as stage I/II (14,42). UC-derived CRCs are usually diagnosed at early stage because of constant clinical surveillance. Indeed, 9 (64%) of our UC-derived CRCs in our cohort were stage 0-I; however, both early-stage (6/9, 66.7%) and later-stage (4/5, 80%) UC-derived CRCs demonstrated EMAST, suggesting that its development likely occurs before advancement of stage (Table 2). In comparing 9 early-stage UC-derived CRCs with 19 early-stage sporadic CRCs, EMAST was significantly more frequent in the UC-derived CRCs (66.7% vs 26.3%, *P* = 0.0426) (Table 2). Interestingly, among the early-stage sporadic CRCs, we identified 1 mononucleotide frameshift for which its presence signifies at least 1 MSI-H tumor that is likely due to hypermethylation of *MLH1* typically seen in up to 15% of sporadic CRCs (1,7). We observed no mononucleotide frameshifts in any of the UC tissues without neoplasia, UC tissues with dysplasia, or UC-derived CRCs (Table 2).

### Inflammation-associated microsatellite alterations (EMAST) are more common with long-standing UC

UC is an inflammatory condition for which neoplasia risk increases over time, particularly after more than 8 years from initial diagnosis (23–26). We wanted to compare the frequency of EMAST between those with short-duration UC and those with long-standing UC (LSUC). We had clinical demographic information on 56 of our 70 samples of patients with UC, and within this cohort, the median disease duration was 15 years (interquartile range 11–19 years). We compared samples of patients with LSUC that had a disease duration of >8 years from diagnosis with samples of patients without LSUC that had a disease duration of ≤8 years. As shown in Table 3, patients with LSUC had an average disease duration of 13 years, whereas patients without LSUC had an average duration of UC of 2 years (*P* < 0.0001). Patients with LSUC were significantly older (average 42 vs 33 years, *P* = 0.0094), but showed no difference in sex composition or extent of colonic disease (left-sided vs pancolitis) (Table 3). Assessing EMAST via our multiplexed PCR, patients with LSUC had significantly higher frequency of EMAST than patients without LSUC (10/25, 40% vs 5/31, 16.1%, *P* = 0.0459) (Table 3). These data suggest progressive accumulation of frameshifts from MSH3 dysfunction with longer duration of UC.

**Table 3.**
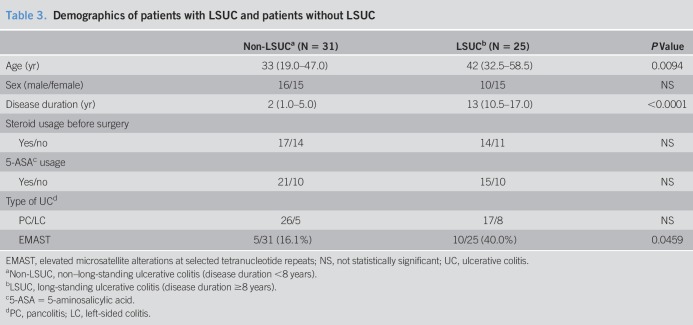
Demographics of patients with LSUC and patients without LSUC

### Between 5% and 10% of microsatellite unstable DNA needs to be present to reliably detect microsatellite frameshifts for application in UC surveillance biopsies

**Figure 3.**
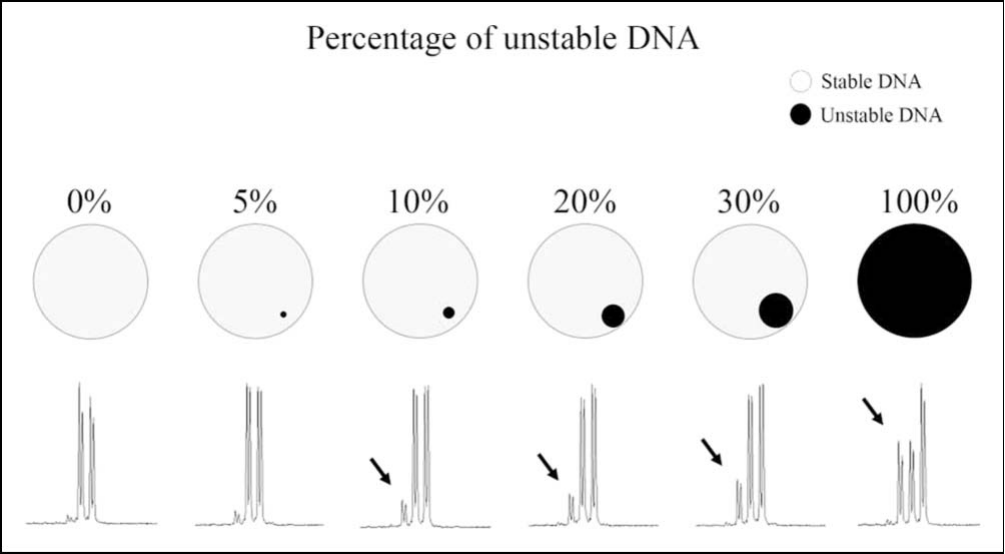
Schematic of admixture of DNA from G5 cells in background of HCT116+3+5 DNA, with fragment analysis at the *D8S321* microsatellite. Arrows indicate increased level of frameshift detected.

Patients with UC are surveyed for dysplasia and/or neoplasia by colonoscopy and protocol-based biopsies from throughout the colon after 8 years of disease duration (24,48). To examine the potential clinical utility of detecting EMAST from UC biopsy material, we (i) determined the approximate mix of frameshifted DNA within non-frameshifted DNA to reliably detect the frameshifts and (ii) assessed the amount of DNA from biopsy forceps taken from resected UC specimens and whether frameshifts could be detected in that amount. We mixed increasing amounts of DNA from G5 cells, which are *MSH3* deficient, in a background of HCT116+3+5 cells, which are MMR proficient (see data from Table 1). Because these 2 cell lines are isogenic, polymorphic allele lengths were generally identical for comparison. As shown in Figure 3 for *D8S321*, a tetranucleotide microsatellite marker, detection of a frameshifted allele could be consistently detected reliably when the amount of frameshifted (or unstable) DNA approached 10% admixture. We measured the DNA amount from standard biopsy forceps taken from 14 biopsies of resected UC specimens. The median amount of DNA obtained from the biopsy forceps was 470.25 ng (interquartile range 232.1–637.5 ng) (see Table, Supplementary Digital Content 3, http://links.lww.com/CTG/A131, which shows individual sample DNA amounts and if sample was detected by fragment analysis). With utilization of our multiplexed PCR method, all samples demonstrated detectable yields at fragment analysis for all mono-, di-, and tetranucleotide markers (see Table, Supplementary Digital Content 3, http://links.lww.com/CTG/A131). Our data indicate that the use of surveillance biopsies for LSUC is feasible for detection of EMAST, with estimation that, at a minimum, between 5% and 10% of endogenous frameshifted DNA is admixed in the sample.

## DISCUSSION

In this study, we determined whether inflammation-associated microsatellite alterations/EMAST are present in the non-neoplastic and proinflammatory cytokine-enriched condition of UC. We demonstrate that (i) the UC epithelium contains cells with reduced nuclear expression of MSH3 (but not MSH6) in the setting of increased IL-6 expression, (ii) UC specimens exhibit inflammation-associated microsatellite alterations/EMAST consistent with MSH3 dysfunction, (iii) inflammation-associated microsatellite alterations/EMAST frequency increases from UC without neoplasia to UC with dysplasia and UC-derived CRCs, (iv) UC-derived CRCs display higher frequency of EMAST compared with early-stage sporadic CRCs, and (v) inflammation-associated microsatellite alterations/EMAST are more frequent within specimens from patients with LSUC (>8 years of duration) than patients with shorter duration. These novel findings signify strong evidence that ongoing DNA mutations occur in the non-neoplastic UC epithelium as a result of inflammation. A limitation of our study is the low number of available UC cases with dysplasia prohibiting differentiation analysis between low-grade and high-grade dysplasia. However, we might expect that EMAST is present in both forms of dysplasia because it is detected in UC samples without dysplasia.

EMAST is a biomarker for MSH3 dysfunction; it does not necessary mean that EMAST itself is causal for progression of neoplasia. In 1 study comparing primary CRC and their matched metastases, the presence of dinucleotide and longer frameshifts was not strikingly different between the primary and its metastasis; however, there were stark differences in LOH events, with LOH at specific sites enriched in metastases (15). This raises the possibility that MSH3's role in repair of DSBs may be the dominant factor in the pathogenesis of metastasis rather than its role in MMR. This hypothesis has not been fully tested for CRCs. In this study, we evaluated whether EMAST was present and operative in UC. We did not examine the presence or consequences of DSBs in UC. Aneuploidy has been observed in UC-derived CRCs and UC dysplastic lesions, and in 1 report, aneuploidy was found in the adjacent nonmalignant mucosa surrounding UC-derived CRCs (49–54). It is tempting to speculate that these observations are a direct result of MSH3 dysfunction, particularly from defective DSB repair. Our findings in this study at least suggest that this is a possibility.

Because EMAST is generated under the influence of proinflammatory cytokines and inflammation, reducing the inflammation would be predictive of reducing the frequency of EMAST (and potentially the future consequence of neoplasia). Epidemiological evidence suggests that prevention of flares and inflammation reduces the risk of future neoplasia in UC (55). In relation to MSI, previous studies only examined mono- and dinucleotide frameshifts, with most studies observing frameshfts at dinucleotide repeats, consistent with potential MSH3 dysfunction rather than any other MMR protein dysfunction (27–40). Although the use of 5-aminosalicylic acid (5-ASA), an anti-inflammatory compound used for mild to moderate UC, failed to reduce the frequency of detected dinucleotide frameshifts after 1 year of usage in patients with UC (56), 5-ASA did reduce frameshifts at mono-, di-, and tetranucleotide microsatellites *in vitro* including reducing the mutant frequency of *TGFBR2* and *ACVR2* (57). Additional studies using biologics might afford an opportunity to evaluate any change in EMAST frequency in UC.

In this study, we ascertained a minimal frameshift mutant admixture for detection of EMAST. We believe that this is important as (i) it will be more reliable than MSH3 IHC in which only 5% of cells show loss of nuclear expression, (ii) most samples from UC colons are taken by biopsy, not resection, and (iii) EMAST could be a potential biomarker for UC and/or UC neoplastic progression in the future. We identified using cell lines that a threshold of between 5% and 10% of microsatellite frameshifted DNA will be needed to reliable detection. Biopsy samples of resected UC colons had adequate amounts of DNA for microsatellite analysis, and all 14 markers demonstrated detectable yields. These data indicate that surveillance biopsies of UC colons could be used for EMAST detection. Potential roles for EMAST in UC management might include (i) as an adjunct in the decision making for more frequent surveillance or decision to surgery with other parameters and (ii) as a biomarker for resolution of inflammation or effectiveness of treatment.

We conclude that the biomarker EMAST is present in non-neoplastic UC colons and represents the biochemical failure by nuclear MSH3 to repair dinucleotide and longer microsatellite sequences. Additional MSH3 DNA repair roles may be abrogated in UC, but we did not test for them in this study. We acknowledge that a limitation of this study is that all of our samples were postsurgery samples, meaning that the UC patient inflammation was severe enough to require surgery. A prospective analysis using biopsies from patients with UC may be needed to more closely approximate typical UC clinical care and surveillance.

## CONFLICTS OF INTEREST

**Guarantor of the article:** John M. Carethers, MD.

**Specific author contributions:** K.M., M. Koi, T.K., S.T.-R., and J.M.C. conceived and designed experiments. K.M., M. Koi, T.K., and S.T.-R. performed experiments. K.M., M. Koi, T.K., S.T.-R., and J.M.C. analyzed data. M.U., H.M., K.K., Y.S., T.M., Y.T., T.Y., M. Mano, E.M., M. Kusunoki, M. Mori, and J.M.C. contributed reagents/materials/analysis tools. K.M., M. Koi, and J.M.C. wrote and edited the manuscript.

**Financial support:** Supported by the United States Public Health Service (NIH grants CA206010, DK067287, and CA162147) and the A. Alfred Taubman Medical Research Institute of the University of Michigan (to J.M.C.). The funders had no role in study design, data collection and analysis, decision to publish, or preparation of the manuscript.

**Potential competing interests:** None to report.

**Study Highlights**

**WHAT IS KNOWN**

✓ EMAST can be generated by the proinflammatory cytokine IL-6 to displace the DNA mismatch repair protein MSH3 from the nucleus to the cytosol causing subsequent di-, tri-, and tetranucleotide frameshift mutations, but this is mainly observed among cancers.
✓ Previous assessment of microsatellite mutations from UC specimens were limited to mono- and dinucleotide repeat markers demonstrating the lack of MSI-H and presumed lack of DNA mismatch repair involvement.

**WHAT IS NEW HERE**

✓ There is loss of function of the DNA mismatch repair protein MSH3 generating di-, tri-, and tetranucleotide frameshift mutations (EMAST) in UC specimens in the absence of neoplasia.
✓ Duration of UC is associated with increased percentage of samples with di-, tri-, and tetranucleotide frameshift mutations.
✓ Increased levels of di-, tri-, and tetranucleotide frameshift mutation function are concomitant with advancement of neoplasia initiated from UC.

**TRANSLATIONAL IMPACT**

✓ Biopsies from patients with UC assayed for EMAST might predict the risk of dysplasia or cancer.
✓ Therapy for UC might reduce EMAST formation and subsequent neoplasia risk.

## Supplementary Material

SUPPLEMENTARY MATERIAL
