# Supplementary material for: Inflammation-Associated Microsatellite Alterations Caused by MSH3 Dysfunction Are Prevalent in Ulcerative Colitis and Increase With Neoplastic Advancement
Source: Clin Transl Gastroenterol. 2019 Nov 26;10(12):e00105. doi: 10.14309/ctg.0000000000000105 (PMC6970556; doi:10.14309/ctg.0000000000000105)
Supplement: SUPPLEMENTARY MATERIAL [file ct9-10-e00105-s001.docx]

**Table, Supplementary Digital Content 1**. PCR primers utilized for multiplex PCR reactions.

| Group | Marker | Fluorescence dyes | Primer sequences | |
| --- | --- | --- | --- | --- |
| Group 1 | BAT25 | HEX | Forward | CTCGCCTCCAAGAATGTAA |
|  |  |  | Reverse | CAGGAAACAGCTATGACTCTGCATTTTAACTATGGCTCTA |
|  | BAT26 | FAM | Forward | TGACTACTTTTGACTTCAGCC |
|  |  |  | Reverse | AACCATTCAACATTTTTAACCC |
|  | D20S85 | NED | Forward | TGGGAGTATCCAGAGAGCTA |
|  |  |  | Reverse | CTCCAGCATGAATTACAGTGTGAGA |
|  | D9S242 | FAM | Forward | TGTAAAACGACGGCCAGTGAGAGTTCCTTCTGGCTTTT |
|  |  |  | Reverse | CAGGAAACAGCTATGACTCCAGTACAAGACTCTGTCAA |
| Group 2 | D2S123 | HEX | Forward | CAGGATGCCTGCCTTTA |
|  |  |  | Reverse | GACTTTCCACCTATGGGA |
|  | D5S346 | HEX | Forward | ACTCACTCTAGTGATAAATCGG |
|  |  |  | Reverse | AGCAGATAAGACAGTATTACTAGTT |
|  | D17S250 | NED | Forward | AAAAGGAAGAATCAAATAGACAATAAA |
|  |  |  | Reverse | CAGCTGGCCATATATATATTTAA |
|  | MYCL1 | FAM | Forward | TGGCGAGACTCCATCAAA |
|  |  |  | Reverse | CTTTTAAGCTGCAACAATTTC |
| Group 3 | D19S394 | HEX | Forward | TGTAAAACGACGGCCAGTGAGACCCTGTCAAAAAAAGAAAG |
|  |  |  | Reverse | TGTCTTCCTGACTACCAGG |
|  | D8S321 | FAM | Forward | TGTAAAACGACGGCCAGTGAAAAGAGTGAGCAAGAGAGAG |
|  |  |  | Reverse | CCCTCCCTCCTTTTCTTTT |
| Group 4 | RBM47 | HEX | Forward | TGAAGGATTGTCACAGAGTTTCA |
|  |  |  | Reverse | AGTTTGGTGGTTGCCATGTT |
|  | D20S82 | FAM | Forward | GAGTGAGACCCTGTCAAAAAAAACA |
|  |  |  | Reverse | ATCTCAGTAGTCAGCCAGTG |
|  | D18S64 | HEX | Forward | TAACTAGAGACAGGCAGAAAA |
|  |  |  | Reverse | AATCAGGAAATCGGCACT |
|  | D18S69 | FAM | Forward | CTCTTTCTCTGACTCTGACC |
|  |  |  | Reverse | GACTTTCTAAGTTCTTGCCAG |
