# Supplementary material for: Inflammation-Associated Microsatellite Alterations Caused by MSH3 Dysfunction Are Prevalent in Ulcerative Colitis and Increase With Neoplastic Advancement
Source: Clin Transl Gastroenterol. 2019 Nov 26;10(12):e00105. doi: 10.14309/ctg.0000000000000105 (PMC6970556; doi:10.14309/ctg.0000000000000105)
Supplement: SUPPLEMENTARY MATERIAL [file ct9-10-e00105-s002.docx]

**Figure, Supplementary Digital Content 2**. **Multiplex PCR for EMAST/ MSI assay**. Examples of fourteen EMAST/ MSI microsatellite markers assayed and subject to fragment analysis. Mononucleotide microsatellites: *BAT25* and *BAT26*; Dinucleotide microsatellites: *D2S123, D5S346, D17S250, D18S64* and *D18S69*, Tetranucleotide microsatellites: *D9S242, D20S82, D20S85, D19S394, D8S321, MYCL1* and *RBM47*.

**
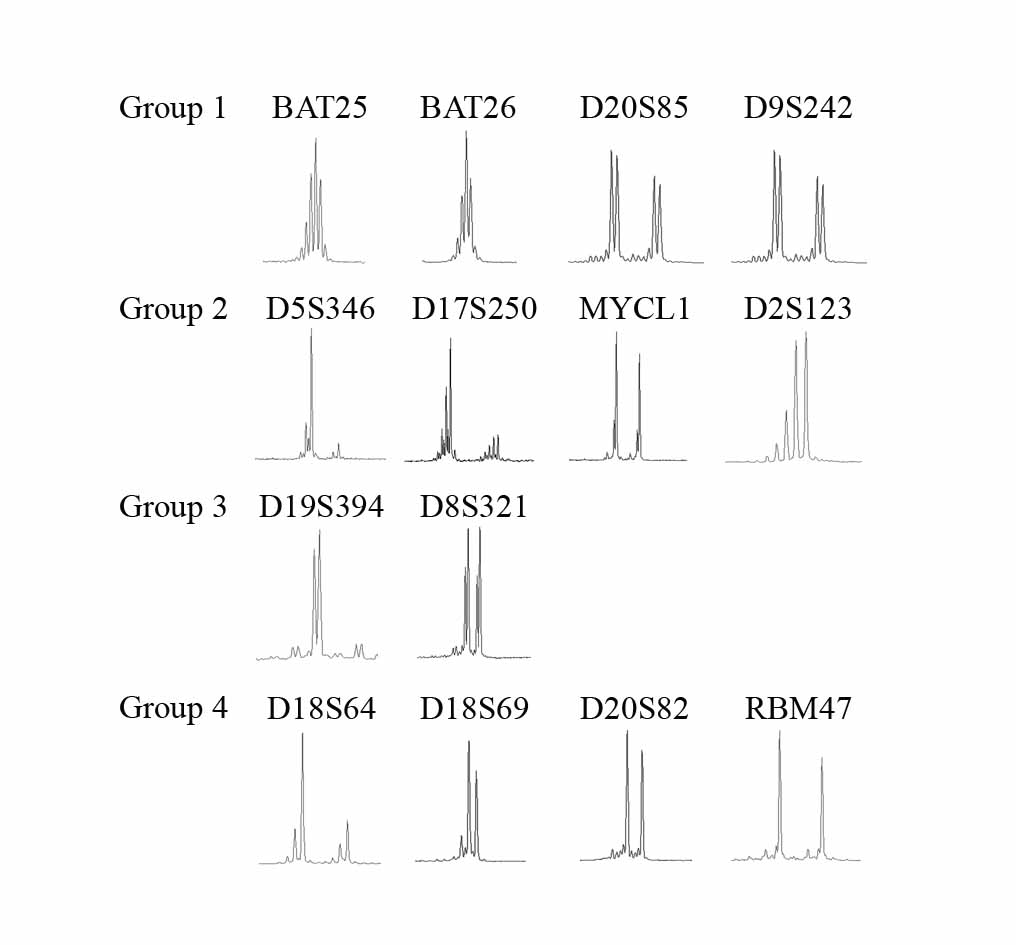
**
