# Supplementary material for: Inflammation-Associated Microsatellite Alterations Caused by MSH3 Dysfunction Are Prevalent in Ulcerative Colitis and Increase With Neoplastic Advancement
Source: Clin Transl Gastroenterol. 2019 Nov 26;10(12):e00105. doi: 10.14309/ctg.0000000000000105 (PMC6970556; doi:10.14309/ctg.0000000000000105)
Supplement: SUPPLEMENTARY MATERIAL [file ct9-10-e00105-s003.docx]

**Table, Supplementary Digital Content 3**. Amount and Quality of DNA from colonoscopy biopsy forceps taken from resected colons.

| Number of sample | Total DNA amount (ng) | Fragment analysis of EMAST/MSI |
| --- | --- | --- |
| 1 | 637.5 | Detected |
| 2 | 85.5 | Detected |
| 3 | 328.8 | Detected |
| 4 | 552.2 | Detected |
| 5 | 222.2 | Detected |
| 6 | 1452.0 | Detected |
| 7 | 304.7 | Detected |
| 8 | 485.1 | Detected |
| 9 | 618.2 | Detected |
| 10 | 99.0 | Detected |
| 11 | 455.4 | Detected |
| 12 | 767.8 | Detected |
| 13 | 1027.4 | Detected |
| 14 | 232.1 | Detected |
| Median  (interquartile range) | 470.25 (232.1-637.5) |  |
